# The Role of Frontline Leaders in Building Health Professional Support for a New Patient Portal: Survey Study

**DOI:** 10.2196/11413

**Published:** 2019-03-22

**Authors:** Sari Kujala, Iiris Hörhammer, Tarja Heponiemi, Kim Josefsson

**Affiliations:** 1 Department of Computer Science Aalto University Espoo Finland; 2 Department of Industrial Engineering and Management Aalto University Espoo Finland; 3 National Institute for Health and Welfare Helsinki Finland

**Keywords:** patient portal, implementation, expectations, organizational readiness, leadership

## Abstract

**Background:**

Effective leadership and change management are thought to contribute to the successful implementation of health information technology innovations. However, limited attention has been paid to the role of frontline leaders in building health professional support for new technical innovations.

**Objective:**

First, we examined whether frontline leaders’ positive expectations of a patient portal and perceptions of its implementation were associated with their support for the portal. Second, we explored whether leaders’ positive perceptions influenced the same unit’s health professional support for the portal.

**Methods:**

Data were collected through an online survey of 2067 health professionals and 401 frontline leaders working in 44 units from 14 health organizations in Finland. The participating organizations run a joint self-care and digital value services project developing a new patient portal for self-management. The survey was conducted before the piloting and implementation of the patient portal.

**Results:**

The frontline leaders’ perception of vision clarity had the strongest association with their own support for the portal (*ß*=.40, *P*<.001). Results also showed an association between leaders’ view of organizational readiness and their support (*ß*=.15, *P*=.04). The leaders’ positive perceptions of the quality of informing about the patient portal was associated with both leaders’ own (*ß*=.16, *P*=.02) and subordinate health professionals’ support for the portal (*ß*=.08, *P*<.001). Furthermore, professional participation in the planning of the portal was positively associated with their support (*ß*=.57, *P*<.001).

**Conclusions:**

Findings suggest that assuring good informing, communicating a clear vision to frontline leaders, and acknowledging organizational readiness for change can increase health professional support for electronic health (eHealth) services in the pre-implementation phase. Results highlight the role of frontline leaders in engaging professionals in the planning and implementation of eHealth services and in building health professionals’ positive attitudes toward the implementation of eHealth services.

## Introduction

Effective leadership and change management are seen as particularly important to the successful implementation of health information technology innovations [1-5]. The leaders’ role in implementation projects can involve mitigating possible risks [6], but leaders can also move proactively to ensure the success of the implementation. For example, Ingebrigtsen et al [7] identified seven categories of leadership behaviors associated with successful outcomes: (1) communicating clear visions and goals, (2) leadership support, (3) establishing a governance structure and coordinating teamwork, (4) arranging training, (5) identifying and appointing champions, (6) addressing work process change, and (7) follow-up. Also, ensuring the ongoing involvement of key stakeholders as well as reserving extra time and reducing workload during the initial implementation are often identified as tasks for leaders [2].

Successful implementation does not only depend on the strategic level of management, but the operational and frontline levels also have their own roles and tasks [7,8]. Implementations often happen in complex organizations, and changes in these environments require a clear vision at all levels [7]. The high-level strategies and objectives need to be integrated with the underlying processes that operationalize the objectives [9]. After all, health care professionals and the attitudes they present are key to preventing resistance and ensuring the active use of new innovations [10,11].

In theory, effective leadership should lead to organizational readiness for change during the pre-implementation stage [12]: those involved are individually and collectively primed, motivated, and technically capable of executing the change [13]. In particular, leaders and change agents need to communicate the benefits of the change. For example, Paré et al [12] found that nurses’ perceptions of vision clarity (why a change is needed) and change appropriateness (that the proposed change is the correct one) had a significant positive influence on organizational readiness in two cross-sectional surveys.

The pre-implementation phase is critical, as the successes and risks tend to accumulate over time [14,15]. Based on their systematic literature review, Ingebrigtsen et al [7] suggest that good communication should spread to all organizational leadership levels before implementation. However, they identified that there is a lack of evidence regarding the role of clinical leaders within different organizational levels and implementation phases.

In this paper, we focus on clinical leaders who are responsible for leadership within an organization that delivers care as defined by Ingebrigtsen et al [7]. In particular, we are interested in frontline leaders such as physicians and nurses working and supervising health professionals in frontline units (eg, wards or care units). These leaders seem to play a critical role in conveying information and motivating health professionals in the early phases of implementation. Although leadership support has been identified as important, it is not known how leaders influence the attitudes of health professionals.

This study aimed to examine whether the health care leaders’ positive expectations of a patient portal and perceptions of its implementation are associated with their own and the subordinate professional support for the portal in the pre-implementation phase. Specifically, leader support for the portal and their perceptions of the portal benefits, the readiness and implementation practices of their units, and the quality of informing about the portal were studied.

## Methods

A survey study was conducted to capture health professional and leader expectations about a national patient portal for self-management that was developed by a self-care and digital value services (*omahoito ja digitaaliset arvopalvelut*, or ODA) project. The data were collected before piloting and implementing the patient portal.

### Study Setting

At the time of the study in spring 2017, the role of information and communication technology (ICT) was widening in Finnish public health care. The objective of the national eHealth (electronic health) and eSocial strategy 2020 is to support the active role of citizens in promoting their own well-being by improving information management and implementing self-management and online services [16]. The aim of the strategy is to support the prevention of health problems, self-assessment of the need for services, and independent coping.

To build online services for citizens, the government has funded an ODA project. The ODA project is run as a joint one comprising 14 municipalities and hospital districts in Finland—including the largest cities of Helsinki, Espoo, Tampere, Turku, and Oulu—totaling 1,766,334 inhabitants in 2014 (32.3% of the Finnish population). The ultimate aim of the ODA project is to provide a national patient portal for self-management and self-service in 2018. In addition to the technical development, particular emphasis has been placed on changing the operational processes by using a participatory approach.

The three main services of the patient portal are patient self-assessment with online well-being coaching programs, a symptom checker for patients to support care navigation and provide information on medical conditions, and a personalized shared care plan tethered to the electronic health records. Using the portal would provide health professionals with additional structured information from patient self-assessment and well-being coaching, symptom checker, and shared care plan. At the time of this study, it was unclear how health professional workflow would be affected. However, the aim was to handle routine tasks automatically, provide patients self-managements tools, and provide more time to personal care.

The ODA project has trained groups of 10 health professionals representing each city and health care region in 2016 and 2017 in a lean process development approach. These groups have planned the operational processes to be supported by the technical solutions in individual services. At the time of the study, none of the organizations had yet started to pilot the services. The first pilot projects started in June 2017, and the whole patient portal entity was planned to start in autumn 2018.

### Questionnaire

There were two versions of the questionnaire, one for professionals and one for their supervisors and leaders. Both surveys collected data on respondent support for the patient portal, background information, respondent’s health organization (city or hospital district), work unit (hospital, health center, etc) and other variables such as efficiency improvements, benefits for patients, the organization’s implementation practices, and the quality of informing. From the professional questionnaire, only the data on their background information and support for a patient portal were included in this study.

The focus of this study was on the leader questionnaire (see Multimedia Appendix 1). The questionnaire was targeted to official supervisors and leaders. Informal leaders such as clinical champions were not included. Five previously validated Information Systems Expectations and Experiences survey items were applied for measuring support for a patient portal [17-19]. An essential part of leader support for a patient portal is that they also support change in work processes. The 5-item organizational readiness and 3-item vision clarity scales [12] were also applied.

In addition, leaders were asked to rate whether they agreed with the positive influences that the new patient portal was planned to have. There were 4 statements about the expected efficiency improvements and 6 statements about the expected benefits for patients. In addition, we asked leaders to evaluate personnel readiness (ie, how willing and able the personnel are to adopt the new portal) using 5 items. They were also asked about the organization’s implementation practices by rating whether they agreed that 9 good implementation practices would take place in the future implementation of the new patient portal. The 9 good implementation practices were identified from the literature [2,6,14,20-23] (see Multimedia Appendix 1). Thus, the content validity of these survey items was established through a literature review [24].

The quality of informing including communicating the goals of the portal was ascertained with 3 questions, and another 3 questions focused on participation in the design of the services. All items were measured on a 5-point Likert-type scale from strongly disagree (1) to strongly agree (5) and included option 6 (I don’t know), which in the statistical analyses was handled as a missing value. User participation was a categorical variable formulated based on 3 questions, showing whether a respondent had participated in the planning of the services of the patient portal.

Before the data were gathered, the content of the questionnaire was reviewed by a group of 3 researchers in the field and the ODA management team. In addition, as recommended [25-27], we tested the reliability of the questionnaire with 4 leaders who completed the questionnaire and talked aloud at the same time about how they understood the questions. Based on the iterative pilot testing, the questionnaire was revised by clarifying wording and slightly modifying some items.

### Data Collection

The data were gathered in the spring of 2017 by using a Web-based questionnaire tool. The project managers of each of the 14 organizations participating in the ODA project sent survey invitations and reminders via email to all health professionals. Based on estimations of the project managers, respondents represented 6.1% of health professionals working in the organizations. As the project managers did not know the number of leaders or how many of the professionals received the survey information, we were not able to calculate the exact response rate. The first page of the questionnaire included a screenshot of one ODA page to illustrate the professional view of the patient portal. To encourage participation, 10 pairs of movie tickets were raffled off among the respondents. The study protocol was reviewed and approved by the Ethical Review Board of Aalto University.

### Analysis

Because the data did not allow linking health professionals with their respective leaders, leader variables were averaged over their organizational unit and assigned to the unit’s professionals. Two small health organizations with units including fewer than two leaders were excluded from the analysis. Units were defined by health organization (city or hospital district) and work unit (hospital, health center, etc). To restrict the analysis to leaders who supervise professionals working with patients, administrative units were excluded from the analyses. The minimum number of professionals in the included units was four.

Descriptive statistics and reliability analyses were performed and mean sum scores were computed for all study variables (see Multimedia Appendix 2). Cronbach alpha scores were all well above .84 (for the leader questioner) and .80 (for the professional questionnaire), indicating good internal consistency [28]. A multicollinearity analysis of the study variables was performed. The variance inflation factors for independent variables in the leader support regression were all below 2.8 and for independent variables in the professional support regression below 5.6, indicating that multicollinearity was not a concern in this study [29].

Associations of leader support with other leader variables were tested with multiple linear regression that included leader support for the patient portal as a dependent variable (DV) and leader age, gender, vision clarity, expected efficiency improvements, expected benefits for patients, personnel readiness, organizational readiness, quality of informing, implementation practices, and participation in the planning of the new patient portal as independent variables (IV).

In the analysis of associations between leader variables and a professional’s support for services, multilevel linear regression was used. This enabled us to control for the natural clustering of employees in organizational units (ie, the dependency between observations of professionals from the same unit). Individual-level professional support was used as the DV; unit-level leader variables and individual-level participation in the planning of the patient portal were used as IVs; and the age of professionals was used as a control variable. Robust estimators for standard errors were used. We first tested the associations of the leader predictors separately using univariate regression. Second, to test the relative contribution of each of the IVs to the total variance of DV explained, we used multiple regression analysis. Predictors that were significant in univariate regression were included in the multiple regression analysis. Backward elimination was used to assess the independence of the predictors. All statistical analyses were performed using Stata version 15.0 (StataCorp LLC).

## Results

### Respondents

Responses from 44 organizational units (eg, primary care in Helsinki) were included in the analysis. All together, 401 leader and 2067 health professional respondents working in these organizational units responded to the questionnaire. The respondents represented 12 health organizations and 6 work units (primary care health center, hospital, psychiatric outpatient clinic, elementary school health care, emergency care, dental care, and other).

Tables 1 and 2 show the background information of the respondents. The majority of leaders (324/401, 80.8%) and professionals (776/2067, 85.92%) were women, and the mean ages of the leaders and professionals were 51.6 and 45.1 years, respectively. The leader respondents were nurses, physicians, counselors, and others whose titles did not reveal profession. A greater proportion of leaders (63/401, 15.7%) than professionals (78/2067, 3.77%) had participated in the planning of the patient portal services.

**Table 1:** Leader background information (n=401).

Characteristic		Value	
**Gender, n (%)**			
	Male		64 (16.0)
	Female		324 (80.8)
	Not reported		13 (3.2)
Age in years, mean (SD)		51.6 (7.9)	
**Profession, n (%)**			
	Nurse leader		177 (44.1)
	Physician leader		94 (23.4)
	Counseling leader		74 (18.5)
	Other		56 (14.0)
Participated in the planning, n (%)		63 (15.7)	
Years of work experience in similar tasks, mean (SD)		10.2 (8.5)	

**Table 2:** Professional background information (n=2067).

Characteristics		Value
**Gender, n (%)**		
	Male	228 (11.0)
	Female	1776 (85.9)
	Not reported	63 (3.1)
Age in years, mean (SD)		45.1 (11.1)
**Profession, n (%)**		
	Hospital nurse	651 (31.5)
	Practical nurse	405 (19.6)
	Doctor/dentist	189 (9.1)
	Public health nurse	167 (8.1)
	Physio and other therapists	124 (6.0)
	Dental nurse	47 (2.3)
	Social worker	42 (2.0)
	Midwife	28 (1.4)
	Administrator	27 (1.3)
	Other	387 (18.7)
Participated in the planning, n (%)		78 (3.7)
Years of work experience in the field, mean (SD)		16.2 (10.8)

### Factors Associated With Leader Support for a New Patient Portal

Multimedia Appendix 2 presents the mean and standard deviation of the study variables. Table 3 presents the results of the multiple linear regression analysis, examining the associations of independent leader variables with the leader support for the patient portal. All IVs except for gender and age were significantly associated with leader support in the univariate analyses (*P*<.001; Multimedia Appendix 3), and therefore they were all included as independent predictors in the multiple regression. Age was included as a control variable as previous studies have suggested associations of age [30] and gender [31] with the use of eHealth technologies.

Results show that the leaders’ clear vision of the future patient portal was moderately associated with their support for the portal (*ß*=.40, *P*<.001). Moreover, good quality of information (*ß*=.16, *P*=.02) and perceived organizational readiness (*ß*=.15, *P*=.04) were modestly positively associated with leader support for the patient portal. In the multiple regression model, expected efficiency improvements, benefits for patients, personnel readiness, and implementation practices did not have a significant independent association with leader support. Overall, the model explained 43% of the variance in leader support for the portal.

### Associations of Unit-Level Leader Factors With Professional Support for the Patient Portal

To test how the perceptions of the leaders were associated with professional support for the patient portal in the pre-implementation phase, we used multilevel modeling. First, in the null model, we tested the effect of clustering on professional support. As indicated in Table 4, the intraclass correlation was 0, indicating that variation in professional support occurred at the individual level rather than at the unit level. Model A includes all potential predictors of professional support that were significant (*P*<.001) in the univariate analyses (Multimedia Appendix 4).

In Model A, only the individual-level variables professional participation in the planning of the services (*ß*=.57, *P*<.001) and professional age (*ß*=.06, *P*=.04) showed statistically significant association with professional support. To further test which independent variables could best explain professional support, we used backward elimination. Model B shows that the individual-level variable professional participation in the planning (*ß*=.57, *P*<.001), unit-level leader view of informing (*ß*=.08, *P*<.001), and individual-level professional age (*ß*=–.06, *P*=.05) alone explain 17% of the variation in professional support with rather modest magnitudes of association. The analysis therefore suggests that there is an association between leader perception of the quality of informing provided and disseminated about the new patient portal and professional support for the portal. The quality of informing variable included aspects of how well the leaders received information and how well the leaders informed their own subordinates. Thus, the results suggest that if the leaders had received information about the portal, they also informed their subordinates. In addition, professionals’ own participation in the planning of the services is associated with their support.

**Table 3:** Multiple linear regression analysis for leader support showing the associations of independent leader variables; *R*^2^=.43.

Variables	*ß*	Standard error	*P* value
Vision clarity	.40	.06	<.001
Efficiency improvements	.04	.07	.58
Benefits for patients	.03	.07	.65
Personnel readiness	.01	.07	.90
Organizational readiness	.15	.07	.04
Quality of informing	.16	.07	.02
Implementation practices	–.08	.07	.25
Participation in the planning (category reference: no participation)	.07	.13	.63
Age	–.05	.04	.30

**Table 4:** Multilevel model of the association of unit-level leader views with professional support (N=1532; 44 units). Continuous variables were used as continuous standardized variables.

Variable		Null model	Model A	Model B
Intercept, regression coefficient (robust standard error)		–0.00 (.03)	–0.03 (.02)	–0.02 (.03)
**Unit-level leader variables, regression coefficient (robust standard error)**				
	Support for services	—	0.01 (.04)	—
	Vision clarity	—	–0.04 (.04)	—
	Expected efficiency improvements	—	0.00 (.04)	—
	Expected benefits for patients	—	0.05 (.06)	—
	Personnel readiness	—	0.01 (.05)	—
	Organizational readiness	—	0.04 (.03)	—
	Quality of informing	—	0.11 (.06)	0.08 (.02)^b^
	Implementation practices	—	–0.08 (.05)	—
**Individual-level variable, regression coefficient (robust standard error)**				
	Professional’s age	—	–0.06 (.03)^a^	–0.06 (.03)
	Professional’s participation (category reference: no participation)	—	0.57 (.10)^b^	0.57 (.10)^b^
*R* ^2^		0.00	0.24	0.17
Rho (intraclass correlation)		0.00	0.00	0.00

^a^*P*<.05.

^b^*P*<.001.

## Discussion

### Principal Findings and Comparison With Prior Work

Effective leadership has been identified as important in the successful implementation of health information technology innovations [1-5]. This survey study aimed at clarifying the role of frontline leaders especially in motivating health professionals in the pre-implementation phase of a patient portal. Frontline leaders’ own support for the new patient portal had strongest association with their perception of vision clarity, information shared in the organization about the portal, and organizational readiness. These factors explained close to half of the variation in leader support, which suggests that special attention should be paid to communicating the vision clearly among the leaders, supporting the communication concerning the new eHealth services in all employee groups, and choosing the timing of implementation wisely to support the organizational readiness. Frontline leaders’ positive perception of quality of information about the patient portal in the organization was also associated with professional support for the portal, which further accentuates the significance of good communication. The multilevel analysis further showed that professionals’ own participation in the planning of the patient portal had positive association with their support for the patient portal. This finding implies that engaging professionals early on in the development can have a notable effect on implementation success.

Overall, the quality of informing was evaluated to be rather low. In the early pre-implementation phase, the health organizations had not yet efficiently informed the personnel that a new patient portal will be implemented. However, informing is important especially in the initiation phase because potential users need to be aware of new services in order to adopt them [20]. Moreover, reaching hundreds or thousands of health professionals takes time. Realistic positive expectations are also known to support later adoption of new services [32].

The findings suggest that good communication and being well informed are particularly important in building frontline leader support for a new eHealth service in the pre-implementation phase. If top management communicates a clear vision and sufficiently conveys information about the new service, frontline leaders are able to inform their subordinate health professionals. During this early implementation phase, the provision of sufficient information about the future patient portal and professional participation in its planning were also positively associated with the health professional support. Thus, frontline leaders have a critical role in engaging health professionals in planning and supporting their positive attitudes, which will in turn ensure active use of the new services [10].

Other study variables were positively associated with leader support in the univariate regression analysis. Most of the study variables were related to implementation practices associated with successful health IT adoption by earlier research [7]. As some of the implementation practices were not yet topical in the pre-implementation phase, the leaders seemed to form expectations based on their previous experiences in their organization. Thus, results suggest that the way technical innovations had previously been implemented influences support for a new service. In addition to implementation practices, change appropriateness regarding how the new portal influences work processes and customers was positively associated with leader support as expected based on earlier research [12].

Ingebrigtsen et al [7] suggest that leaders at all levels of the organization support successful IT adoption. For example, all leadership levels should communicate a clear vision and the goals of the new system. Our research results suggest that frontline leaders are more prepared to support the new patient portal and communicate a clear vision to their subordinates if they have received information about the vision from the upper levels. As the strategic level is not in direct contact with health professionals, it is the frontline level that has a critical role in communicating the vision and providing leadership support. On the other hand, the frontline needs information and resources from the upper levels to be effective.

In summary, the results support earlier research findings. The contribution of our study is that it clarifies the role of the frontline leaders in the early phases of implementing a new patient portal. Our results highlight the importance of vision clarity and conveying information to the frontline leaders so they can create support among health professionals for a new patient portal. In large and complex organizations, frontline leaders have a critical role in communicating and spreading the vision for the health professionals, engaging them in planning and supporting positive attitudes among the professionals.

### Limitations

In this study, we focused on the pre-implementation phase and a specific application, the patient portal for self-management. Due to the cross-sectional study design, it is not possible to infer causality relationships between variables. Future longitudinal studies should clarify the role of frontline leaders’ pre-implementation perceptions in the later phases of implementation. The importance of top management support has been highlighted in earlier research [5] but was not directly studied here.

Furthermore, the variable quality of informing was not one-dimensional as it embedded aspects on how well the leaders received information and how well the leaders informed their own subordinates. The Cronbach alpha of the measure was very high (.92) supporting the assumption that the frontline leaders cannot inform their subordinates if they have not received the information from the organization.

We were not able to link leaders specifically with their subordinate health professionals. Instead, we averaged leader variables over the unit they worked in. However, the statistically significant association found between leader and professional variables in our study can be considered strong, since only averaged values of leader variables over a work unit could be used in the analysis. It is possible that associations would have been stronger and weaker associations could have been found if individual leaders could have been linked to their respective subordinates.

## Appendix

Multimedia Appendix 1 Questionnaire.

Multimedia Appendix 2 Mean and standard deviation of the key leader variables.

Multimedia Appendix 3 Univariate regression analyses of the leader variables associated with leader support.

Multimedia Appendix 4 Univariate regression analyses of the leader variables associated with professional support.

## Abbreviations

DV: dependent variable
eHealth: electronic health
ICT: information and communication technology
IV: independent variable
ODA: omahoito ja digitaaliset arvopalvelut
