# Supplementary material for: The Role of Frontline Leaders in Building Health Professional Support for a New Patient Portal: Survey Study
Source: J Med Internet Res. 2019 Mar 22;21(3):e11413. doi: 10.2196/11413 (PMC6450477; doi:10.2196/11413)
Supplement: Multimedia Appendix 1 [file jmir_v21i3e11413_app1.pdf]

APPENDIX 1: The questionnaire

Expectation questionnaire

The scales ranged from 1 (strongly disagree) to 5 (strongly agree) and included option 6 (I don't know).

QUALITY OF INFORMING
What is your opinion about the information received regarding the future ODA portal?

1. I have received enough information about the ODA portal.
2. I have informed my subordinates about the ODA portal.
3. The goals of the ODA portal have been provided to our unit.

SUPPORT
What is your opinion about the implementation of the ODA portal?

1. I support the implementation of the ODA portal.
2. My co-workers support the implementation of the ODA portal.
3. My leader supports the implementation of the ODA portal.
4. I understand the organization's decision to implement the ODA portal.
5. I am committed to supporting the functional change required by the ODA portal.

VISION CLARITY
Please evaluate the future ODA portal in your unit:

1. The ODA portal is important in realizing our strategy.
2. I believe there are legitimate reasons for us to introduce the ODA portal in our unit.
3. We definitely need new ODA tools to improve the way we work around here.

EFFICIENCY IMPROVEMENTS
Please evaluate the influence of the ODA portal on your unit's functioning:

1. Using the ODA portal will be cost-efficient in my unit.
2. The ODA portal will reduce the amount of routine work.
3. The ODA portal can be well integrated with existing information systems.
4. The ODA portal will be suitable for our local conditions.

BENEFITS FOR PATIENTS
Please evaluate the influence of the ODA portal on your patients:

1. I believe that by using the ODA portal, patients will have the same criteria to receive care/service.
2. I believe that the ODA portal will support self-management and self-service.
3. I believe that the ODA portal will support individual care/service.
4. I believe that the ODA portal will support orderliness in patient care and service.
5. I believe that more than half of the patients are willing to use the ODA portal.
6. I believe that more than half of the patients are capable of using the ODA portal.

What kind of utility do you expect the ODA portal to provide to your unit or your own work? (Open question)

What challenges do you expect the ODA portal to cause to your unit or to your work? (Open question)

ORGANIZATIONAL READINESS
Please evaluate the future ODA portal in your unit:
1. I believe the ODA portal can be successfully implemented in our unit.
2. The deployment of the ODA portal should be delayed.
3. The deployment of the ODA portal in our unit is timely.
4. Our unit is ready to take on this technological change.
5. Our unit is ready to take on this functional change.

IMPLEMENTATION PRACTICES
Please evaluate the implementation practices of the ODA portal in your unit?
1. We have set goals for the implementation of the ODA portal
2. We have set measures for following the implementation of the ODA portal.
3. The plan for measuring the benefits of the ODA portal is ready.
4. My unit has a person who is responsible for the implementation of the ODA portal.
5. My unit has a person who encourages others to use the ODA portal.
6. The personnel have an opportunity to participate in planning the ODA portal.
7. There will be enough training for using the ODA portal.
8. In the event of problems, there will be enough technical support.
9. The personnel will be allowed to have separate working time for adopting the ODA portal.

EFFECT ON WORK PROCESSES
How will the ODA portal influence the current work processes? (Multiple-choice question)
1. The work processes will be planned again.
2. The new ODA portal will complement the current work processes.
3. The current work processes will not be changed.
4. Something else (please specify):

PERSONNEL READINESS
Please evaluate the readiness of the personnel in your unit to adopt the ODA portal:
1. The personnel know the aimed benefits of the ODA portal.
2. The personnel accept the implementation of the ODA portal.
3. The personnel are willing to tell patients about the ODA portal.
4. The personnel have enough computer skills to use the ODA portal.
5. The personnel know how their tasks will change with the ODA portal.

USER PARTICIPATION
Have you participated in planning the ODA portal? (Options: Yes, No)
1. The ODA training of operational change
2. The ODA working groups
3. Internal planning in my unit
4. Something else (please specify):

If you wish you can comment on or express requests for the implementation of the ODA portal: (Open question)

Background questions

1. How many years have you worked in your current field?
2. Gender
3. Age
4. Job title
5. City or health care area
6. Your primary workplace
