# Supplementary material for: The Role of Frontline Leaders in Building Health Professional Support for a New Patient Portal: Survey Study
Source: J Med Internet Res. 2019 Mar 22;21(3):e11413. doi: 10.2196/11413 (PMC6450477; doi:10.2196/11413)
Supplement: Multimedia Appendix 2 [file jmir_v21i3e11413_app2.pdf]

APPENDIX 2: Mean and S.D. of the key leader variables.

Variables	Mean	S.D.
Support for services	4.2	.79
Vision clarity	4.1	.90
Expected benefits for patients	3.6	.86
Expected efficiency improvements	3.5	.89
Organizational readiness	3.3	.88
Personnel readiness	2.8	1.0
Quality of informing	2.3	1.3
Implementation practices	2.2	1.2

The scales of the variables were ranging from 1(fully disagree) to 5 (fully agree) and included also option 6 (I don't know) that was removed from the analysis.
