# Supplementary material for: The Role of Frontline Leaders in Building Health Professional Support for a New Patient Portal: Survey Study
Source: J Med Internet Res. 2019 Mar 22;21(3):e11413. doi: 10.2196/11413 (PMC6450477; doi:10.2196/11413)
Supplement: Multimedia Appendix 3 [file jmir_v21i3e11413_app3.pdf]

APPENDIX 3: Univariate regression analyses of the leader variables associated with leader support.

Leader variable	Correlation coefficient	Standard Error	P	95% CI
Support for services				
Vision clarity	0.56	0.04	<.001	0.48 - 0.64
Efficiency improvements	0.46	0.04	<.001	0.38 - 0.55
Benefits for patients	0.49	0.04	<.001	0.41 - 0.58
Personnel readiness	0.34	0.05	<.001	0.25 - 0.44
Organizational readiness	0.54	0.04	<.001	0.46 - 0.63
Information	0.38	0.05	<.001	0.28 - 0.47
Implementation practices	0.37	0.05	<.001	0.27 - 0.46
Age	-0.08	0.05	.12	-0.19 - 0.02
Gender (category reference: female)	-0.04	0.15	.80	-0.32 - 0.25

Note: Continuous variables were used as continuous standardized variables.
