# Supplementary material for: The Role of Frontline Leaders in Building Health Professional Support for a New Patient Portal: Survey Study
Source: J Med Internet Res. 2019 Mar 22;21(3):e11413. doi: 10.2196/11413 (PMC6450477; doi:10.2196/11413)
Supplement: Multimedia Appendix 4 [file jmir_v21i3e11413_app4.pdf]

APPENDIX 4: Univariate regression analyses of the leader variables associated with professional support.

Leader variable	Correlation coefficient	Standard Error	P	95% CI
Support for services	0.07	0.03	.003	0.02 - 0.12
Vision clarity	0.06	0.03	.030	0.01 - 0.11
Efficiency improvements	0.06	0.03	.014	0.01 - 0.11
Benefits for patients	0.09	0.03	.001	0.04 - 0.14
Personnel readiness	0.07	0.03	.004	0.02 - 0.12
Organizational readiness	0.08	0.03	.003	0.03 - 0.13
Information	0.09	0.02	.001	0.07 - 0.13
Implementation practices	0.06	0.03	.012	0.01 - 0.11

Note: Continuous variables were used as continuous standardized variables.
